# Supplementary material for: Novel Driver Strength Index highlights important cancer genes in TCGA PanCanAtlas patients
Source: PeerJ. 2022 Aug 11;10:e13860. doi: 10.7717/peerj.13860 (PMC9375969; doi:10.7717/peerj.13860)
Supplement: Supplemental Information 2 [file peerj-10-13860-s002.zip › COHORTS/genes plots/age/2021_8_16_14_9_distribution_cohorts_65-69.pdf]

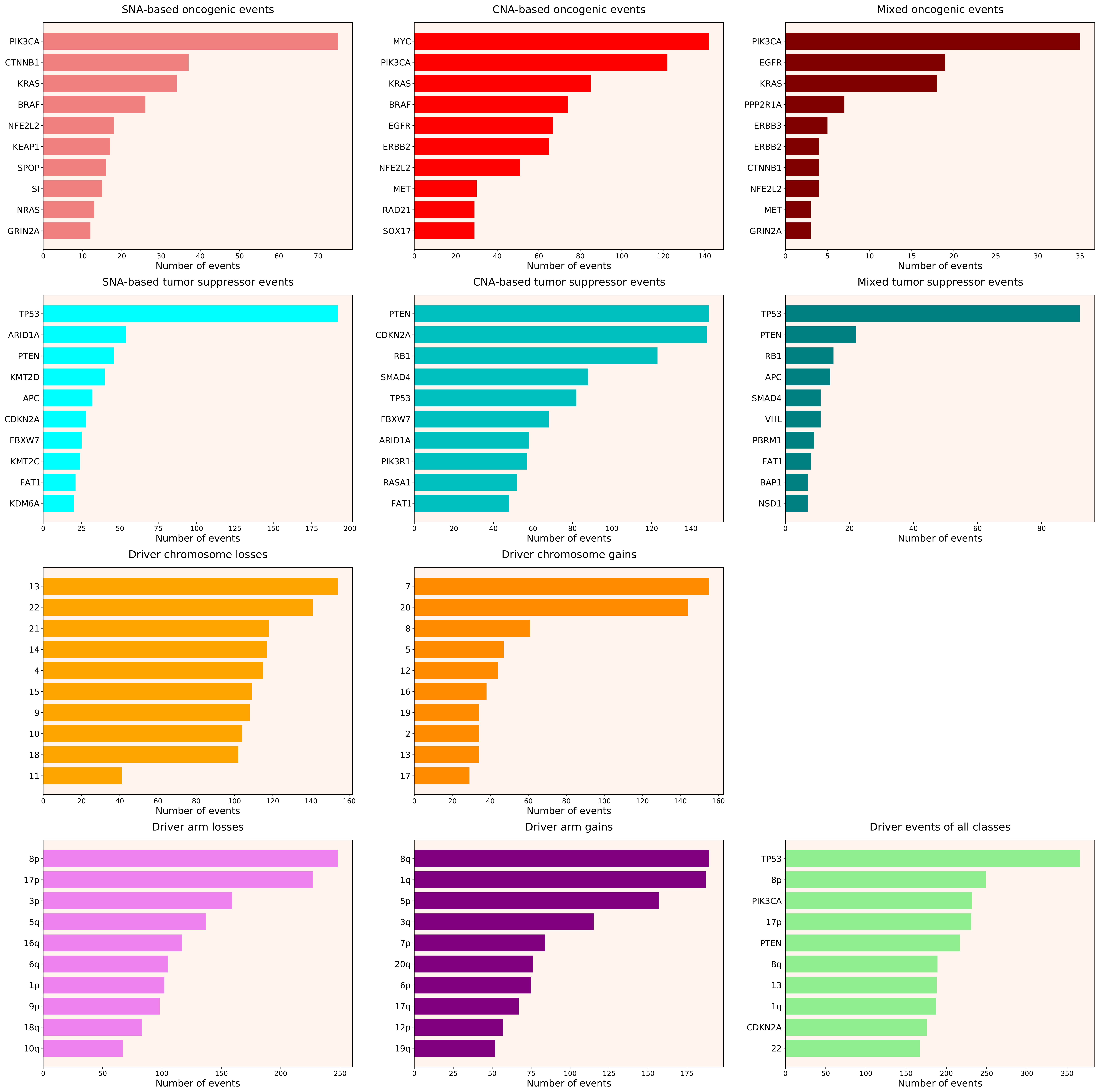
SNA-based oncogenic events
PIK3CA
CTNNB1
KRAS
BRAF
NFE2L2
KEAP1
SPOP
SI
NRAS
GRIN2A
Number of events
CNA-based oncogenic events
MYC
PIK3CA
KRAS
BRAF
EGFR
ERBB2
NFE2L2
MET
RAD21
SOX17
Number of events
Mixed oncogenic events
PIK3CA
EGFR
KRAS
PPP2R1A
ERBB3
ERBB2
CTNNB1
NFE2L2
MET
GRIN2A
Number of events
SNA-based tumor suppressor events
TP53
ARID1A
PTEN
KMT2D
APC
CDKN2A
FBXW7
KMT2C
FAT1
KDM6A
Number of events
CNA-based tumor suppressor events
PTEN
CDKN2A
RB1
SMAD4
TP53
FBXW7
ARID1A
PIK3R1
RASA1
FAT1
Number of events
Mixed tumor suppressor events
TP53
PTEN
RB1
APC
SMAD4
VHL
PBRM1
FAT1
BAP1
NSD1
Number of events
Driver chromosome losses
13
22
21
14
4
15
9
10
18
11
Number of events
Driver chromosome gains
7
20
8
5
12
16
19
2
13
17
Number of events
Driver arm losses
8p
17p
3p
5q
16q
6q
1p
9p
18q
10q
Number of events
Driver arm gains
8q
1q
5p
3q
7p
20q
6p
17q
12p
19q
Number of events
Driver events of all classes
TP53
8p
PIK3CA
17p
PTEN
8q
13
1q
CDKN2A
22
Number of events
